# Beautiful swimmers attack at low tide

**DOI:** 10.1002/ecy.3787

**Published:** 2022-08-01

**Authors:** David S. Johnson

**Affiliations:** ^1^ Virginia Institute of Marine Science Gloucester Point Virginia USA

**Keywords:** ambush predator, blue crabs, *Callinectes sapidus*, fiddler crabs, *Sesarma reticulatum*, sit‐and‐wait, *Uca pugnax*

It is low tide in a salt marsh. A blue crab, *Callinectes sapidus*, digs a shallow‐water pit. It appears that the crab has missed the outgoing tide and is waiting for the next one. But it is not the tide the crab is waiting for. A female fiddler crab, *Minuca pugnax*, picks at the mud near the pit with its claws. It is eating. The blue crab waits. The fiddler crab moves closer to the pit. Suddenly, the water explodes as the blue crab seizes the fiddler crab and pulls it into the pit. Now it is the blue crab's turn to eat.

While conducting research in a Virginia salt marsh at low tide in September 2021, colleagues and I saw blue crabs ambushing fiddler crabs from shallow, water‐filled pits (Figure 1; Video S1), like crocodiles ambushing wildebeests in Africa. Two colleagues saw a blue crab fleeing through the grass with a purple marsh crab, *Sesarma reticulatum*, one of its prey, in its claw (Cora Baird, Leah Scott, personal communication). One blue crab emerged from a pit, crawled slowly across the mud with its body low to the ground, stalked a fiddler crab, snatched it, and ran back to its pit to eat. The large claws of male fiddler crabs littered the outside of several blue‐crab pits (Figure 2), like the discarded bones of villagers outside a dragon's lair. We had discovered a predator—one that lives, breathes, and hunts underwater—feeding out of the water. The blue crab did this using pockets of water left behind by the tide to wet its gills and hide its body. I found one blue crab as far as 70 m (almost 800 body lengths) into the marsh nestled in its own tiny sea among the grass.

**FIGURE 1:**
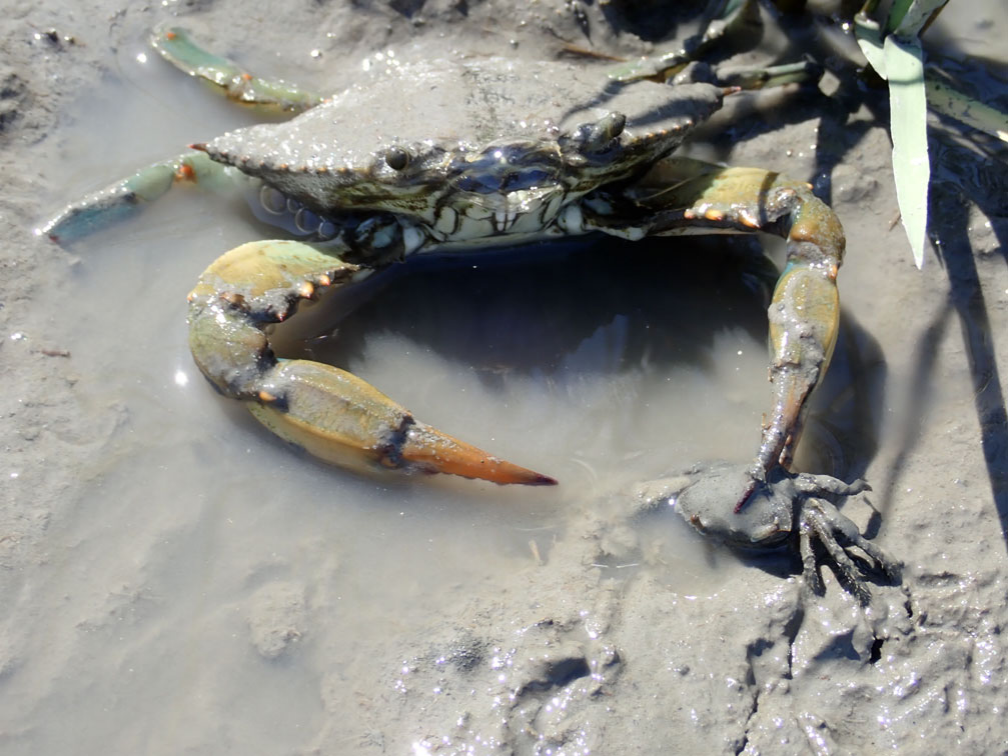
A blue crab with a recently caught fiddler crab.

**FIGURE 2:**
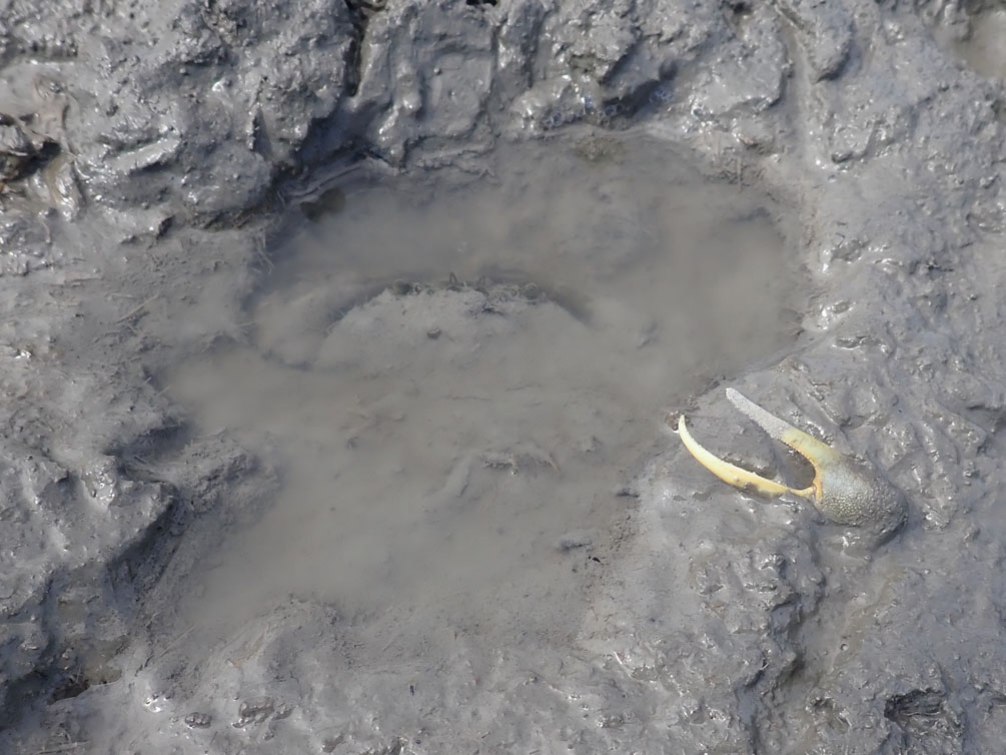
The major claw of a male fiddler crab lays next to the pit of a blue crab, which waits inside.

These observations challenge two well known paradigms of intertidal ecology. First, aquatic predators such as blue crabs feed in the intertidal only during high tide (Fitz & Wiegert, 1991). Second, semiterrestrial crabs, such as fiddler crabs and purple marsh crabs, are safe from aquatic predators because they are active at low tide and retreat to their burrows at high tide (Christy, 1982; Cannicci et al., 1996). That said, blue crabs are known to dash at least 1 m onto land to snatch fiddler crabs before returning to the water to dismember and eat them (Herrnkind, 1968; Hughes & Seed, 1981). But the behavior we saw was different. Blue crabs were not chasing their prey on land; they were waiting on land for their prey to come to them.

I found no reports of these ambush‐style attacks from pits at low tide by blue crabs or other portunid (also known as swimming) crabs in the literature or among my colleagues except for one who said they would seen similar attacks in Georgia and Mississippi salt marshes (Richard Heard, personal communication). Perhaps blue crabs use this hunting style throughout their range (western Atlantic, Europe, and Asia; Johnson, 2015; Mancinelli et al., 2021). More generally, perhaps other portunid crabs use these ambush‐style attacks at low tide as well. For instance, the mangrove swimming crab, *Thalamita crenata*, which is found throughout the Indo‐Pacific, shelters in small pools in the mangroves at low tide (Cannicci et al., 1996). It, too, eats fiddler and sesarmid crabs (Cannicci et al., 1996). As for *C. sapidus*, it may also ambush prey from shallow pools at low tide. Future studies using camera or binocular surveys would reveal if other aquatic crab species are hunting prey at low tide.

We know that blue crabs feed in salt marshes at high tide (Fitz & Wiegert, 1991), but we do not know how many feed at low tide. Is ambush hunting at low tide effective? To answer these questions, I returned to the same marsh 2 weeks after our first observations to record blue‐crab densities, sizes, and attacks. Measurements and observations were taken at low tide on a warm (29°C), sunny day (please refer to Appendix S1 for detailed methods). Densities ranged from 0 to 0.6 m^−2^, depending on the area sampled (Appendix S1: Figure S1a). Most (83%) of the crabs were juveniles (<10 cm carapace width) with carapace widths ranging from 6.0 to 11.8 cm (*n* = 31) (Appendix S1: Figure S1b). The pits were typically not much wider or deeper than the blue crabs (mean width (*n* = 31): 19 cm, range 3–36 cm; mean depth (*n* = 31): 3.4 cm, range: 0.5–8.0 cm). This result suggests that blue crabs are digging the pits themselves. Video evidence supports this suggestion, as it captured blue crabs burying in pits or scooping mud out with their claws (Video S1). Blue crabs were not loyal to their pit and would move into an empty pit (or a water‐filled bootprint) and evict another blue crab if necessary (Video S1).

Out of the 33 attacks captured on 37 h of video, 11 (33% or one out of three) were successful. Most attacks happened when a fiddler crab was in front of the blue crab, but occasionally when a fiddler crab was behind or beside the blue crab. Blue crabs did not always attack, however, even when fiddler crabs fed at the edge of the pits. It is unclear what motivated the blue crabs to attack. Based on videos, I estimated that blue crabs chased fiddler crabs up to 5 cm if they missed on the first strike. Blue crabs sometimes tried to steal fiddler crabs from each other (Video S1).

Blue crabs feeding at low tide offer exciting opportunities to study predator–prey interactions regarding predation risk and foraging trade‐offs. For instance, low‐tide feeding by blue crabs escalates the risk of attacks by bird that also feed at low tide. However, a laughing gull, *Leucophaeus atricilla*, a blue‐crab predator, walked within centimeters of a blue crab in a pit, but did not appear to notice it (Video S1). When in their pits, blue crabs remain still and are camouflaged with mud (Appendix S1: Figure S2a). I hypothesize that this camouflage and motionless behavior protects them from bird predators. Future studies can test this hypothesis rigorously with tethering studies combined with video surveillance and behavioral observations.

My colleagues and I observed blue crabs ambushing low‐tide prey while conducting research on purple marsh crabs, which created denuded areas in the salt marsh through their overgrazing of *Spartina alterniflora*. I hypothesize that purple marsh crabs help blue crabs feed in the marsh at low tide, directly as prey and indirectly by removing plants. For instance, there were almost twice as many blue crabs in areas where plants had been removed by purple marsh crab grazing compared with in the areas with plants (Appendix S1: Figure S1a). It may be easier for blue crabs to dig pits and catch fiddler crabs when plants are removed. Fiddler crab densities are typically higher in areas denuded of grass by purple marsh crabs compared with in areas with grass (Williams & Johnson, 2021). If purple marsh crabs do help blue crabs hunt in the marsh at low tide, then blue crabs should be more abundant in marshes where purple marsh‐crab grazing is substantial versus marshes where it is limited. Conducting surveys at low tide for blue crabs in marshes with and without substantial purple marsh crab grazing would help answer this question.

Salt marshes are rich with prey that fuel marine predators; however, the marsh surface is only available to them part of the time, when it is flooded. The blue crab seems to have solved this problem with a feeding strategy that allows for 24‐h feeding. This suggests that salt marshes are more important to their production than previously thought (Hyman et al., 2022; Lipcius et al., 2005). The blue‐crab fishery contributes (US)$100s of millions of dollars to the United States economy. My observations underscore how vital salt marshes are to this fishery (Baker et al., 2020; Hyman et al., 2022). To better estimate just how vital they are to the blue‐crab fishery, future studies should consider stable isotope analysis combined with gut content analysis of blue crabs and population estimates in salt marshes at high and low tides.

Predators can link different ecosystems. Blue crabs feeding in salt marshes at low tide offer the fascinating opportunity to study how predator behavior can affect trophic subsidies (the movement of energy from one ecosystem to another; Lesser et al., 2021). Trophic subsidies are often seen as the passive movement of energy (e.g., dead organic matter floating downstream, Odum, 1980), but predators can move energy between ecosystems (Nelson et al., 2019). To know how strongly ecosystems are linked, we must first know when and where predators are crossing boundaries to feed. Just as the crocodile links the river to the savanna, the blue crab connects the estuary to the salt marsh.

## CONFLICT OF INTEREST

The author declares no conflict of interest.

## Supporting information


Appendix S1

Video S1

Video S1 Legend

## Data Availability

Data (Johnson, 2022) are available from the Environmental Data Initiative at https://doi.org/10.6073/pasta/acabd51fc75a744d7d97072d950decd6.
