# Supplementary material for: Beautiful swimmers attack at low tide
Source: Ecology. 2022 Aug 1;103(10):e3787. doi: 10.1002/ecy.3787 (PMC9786347; doi:10.1002/ecy.3787)
Supplement: Supplementary file 1 — Appendix S1 [file ECY-103-e3787-s002.pdf]

Appendix S1

Site description
These observations were made in salt marshes in Painter, Virginia (37° 31' 1" N 75° 47' 06" W). These marshes are on the Atlantic side of the Eastern Shore of Virginia and along the tidally-influenced portion of the Machipongo River.

Methods for surveys
Based on previous observations, blue crabs were found in three zones of the marsh (Figure Appendix S1: S2C). 1) The low marsh, which I defined as having tall-form *Spartina alterniflora* and being adjacent to the head of tidal creeks. 2) A 10 – 20 m wide band of bare mud denuded by grazing by the purple marsh crab *Sesarma reticulatum*. 3) The high marsh, which I defined as having short- form S. *alterniflora* and was at a higher elevation than the low marsh and denuded zone.

To estimate the density of blue crabs in pits in each zone, I haphazardly tossed a 1 m^2 quadrat 15 times in each zone along a 200 m transect. To estimate crab body size and pit size, I measured the carapace width of the 1st 15 crabs I encountered in each zone and their corresponding pit width (the longest dimension of the pit) and depth at center. I only found one blue crab in the high marsh, which was outside of the quadrats. I also noted the sex of each crab.

I do not think tossing the quadrats affected my estimates of blue crabs in pits. When I saw quadrats land, I saw fiddler crabs scatter, but not blue crabs. Further, blue crabs did not flee from their pits when colleagues and I walked next to them. Sometimes we stepped on top of them before they moved. Blue crabs seem to stay in place when people, quadrats, and birds are around.

To record blue-crab attacks, I attached action cameras (SJCam (SJCam Limited, Shenzhen, China,) and Campak (Campak Inc., Livingston, New Jersey, USA) to PVC poles 0.5 – 1.5 m above the sediment surface primarily in the denuded zones because it was easier to see the crabs without plants. Density estimates, measurements, and recordings were made at low tide between 09:00 and 13:00 on 28 September 2021. Peak low tide occurred at approximately 08:00 and the tide did not cover the marsh again until after measurements and recordings were taken. It was a warm (29°C at noon), sunny day.

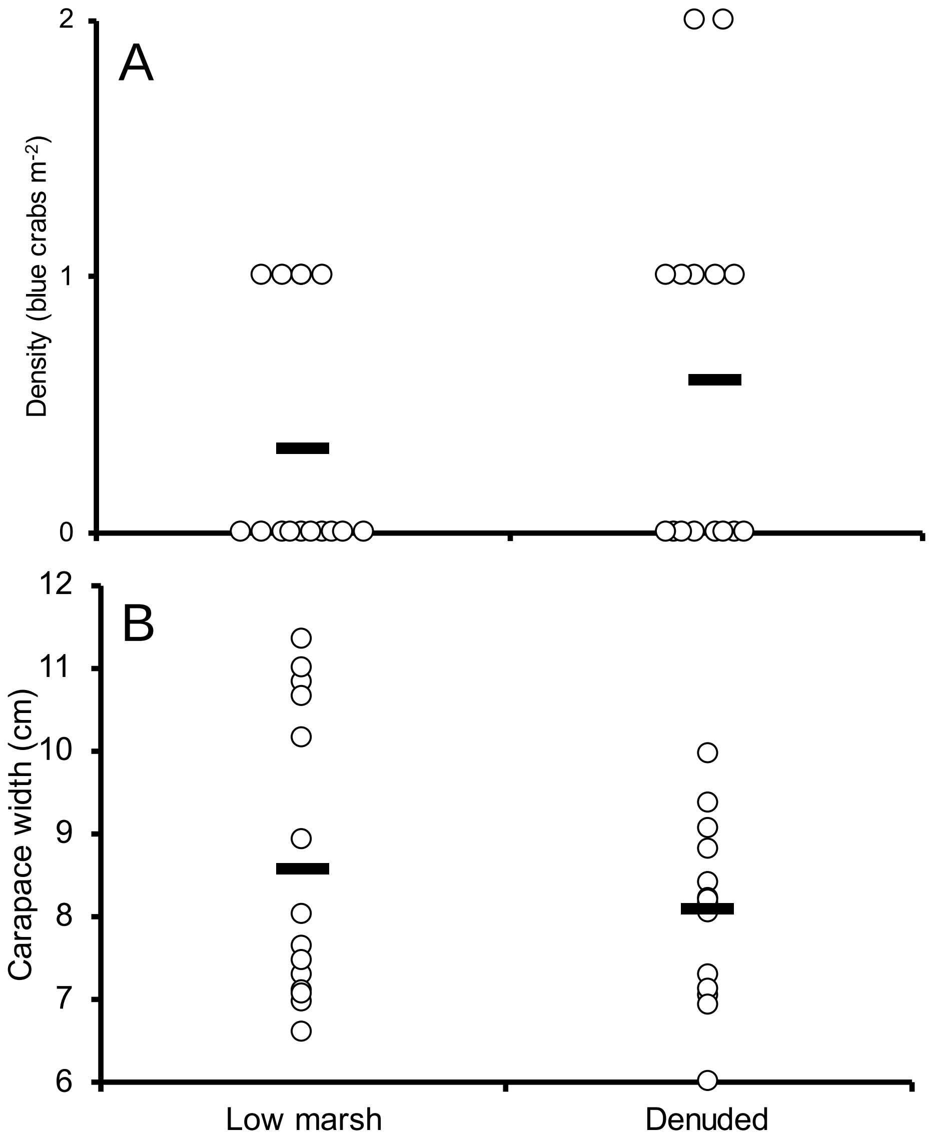

Figure S1: A) Density (# m^{-2}) of blue crabs found in pits in the low marsh and denuded zones from haphazardly tossed quadrats (n=15). A density of 0 crabs m^{-2} was estimated for the high marsh based on this method, although they are present this part of the marsh. B) Carapace width of the blue crabs found in pits in the low marsh and denuded zones of the salt marsh (n=15). One blue crab was found in the high marsh but is not displayed (carapace width=6.8 cm). O = raw data, ▬ = mean.

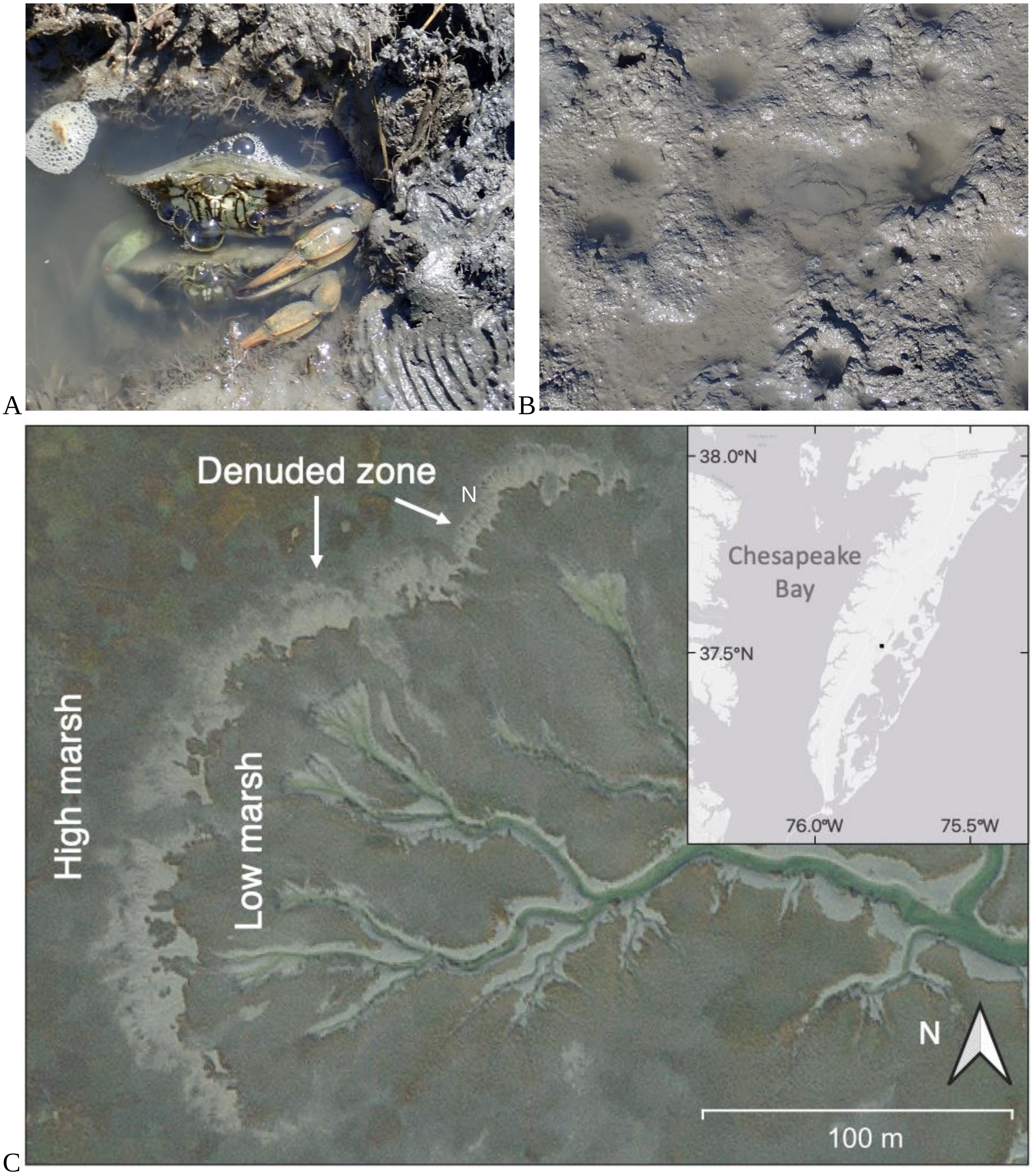

Figure S2.
A. Blue crabs in a pre- or post-copulatory embrace (also known as ‘doublers’) with the male on top and female on bottom. Males cradle carry females before she molts (pre-copulatory embrace), mates with her once she molts, and then cradle carries her after mating (post-copulatory embrace) until her exoskeleton hardens. Females only mate once.
B. A top-side view of a blue crab that is well camouflaged in its pit. It sits among fiddler-crab burrows. The outline of the blue crab’s rostrum and right chela can be seen in the middle of the picture.

C. Inset: Map of the Eastern Shore of Virginia. Black square indicates the location of the salt marsh studied here.
Detailed image: Orthoimage of tidal creeks and salt marsh in Painter, Virginia, where blue crab attacks were observed. The low marsh is dominated by tall-form *Spartina alterniflora*, high marsh dominated by short-form *S. alterniflora*. The denuded zones are swaths of bare mud where grass has been overgrazed by the purple marsh crab, *Sesarma reticulatum*. Orthoimage is from October 2018, the most recent available, but it still shows the same plant patterns in the marsh we see today. High resolution orthoimagery courtesy of the National Agriculture Imagery Program, courtesy of United States Geologic Survey via EarthExplore.
