# Supplementary material for: Beautiful swimmers attack at low tide
Source: Ecology. 2022 Aug 1;103(10):e3787. doi: 10.1002/ecy.3787 (PMC9786347; doi:10.1002/ecy.3787)
Supplement: Supplementary file 3 — Video S1 Legend [file ECY-103-e3787-s003.pdf]

D.S. Johnson, Beautiful swimmers attack at low tide, *Ecology*

Video S1 Legend

Videos recording blue crab behavior in salt marsh at low tide. These videos were taken at low tide on 28 September 2021 in Painter, Virginia. I used action cameras (SJCam (SJCam Limited, Shenzhen, China,) and Campak (Campak Inc., Livingston, New Jersey, USA) attached to PVC poles 0.5–1.5 m above the sediment surface primarily in the denuded zones because it was easier to see the crabs without plants. It was a warm (29°C at noon), sunny day.

Some video segments have been edited in the following ways to highlight behaviors: zooming in, slow-motion, and red arrows. Video has been reduced from original resolution to reduce file size.

Videographer: David S. Johnson
Video Editor: Anne Turner
